# Global health emergencies during the pandemic and their solutions

**DOI:** 10.3906/sag-2106-183

**Published:** 2021-12-17

**Authors:** İrfan ŞENCAN, Dilek BULUT, İsmail Hakkı ŞENCAN, Canan AĞALAR

**Affiliations:** 1 Department of Infectious Diseases and Clinical Microbiology, Health Sciences University, Dışkapı Yıldırım Beyazıt Teaching and Research Hospital, Ankara Turkey; 2 Department of General Surgery, Health Sciences University, Ankara Teaching and Research Hospital, Ankara Turkey; 3 Department of Infectious Diseases and Clinical Microbiology, Fenerbahçe University, Medicana Ataşehir Hospital, İstanbul Turkey

**Keywords:** Covid-19, health system, emergency, crisis, vulnerability, durability

## Abstract

In this review, we evaluated health care problems, which were not common before pandemic outbreak but have been common issues after its appereance and approaches to control pandemic considering its influences on people. We revised current health care developing approaches under the light of experience obtained throughout the pandemic so far. The aim is to be prepared in advance for possible upcoming pandemics. As in Covid 19 pandemics, such long lasting and widely affecting situations, durability is also very important together with flexibility and quickness. To provide durability, we need global policies taking the health to its center as well as health system policies.

## 1. Introduction

Covid-19, the new strain of human coronavirus emerged in Wuhan city, China, was officially identified as a pandemic by the World Health Organisation (WHO) upon receiving breakout reports from nearly every continent [1,2]. Throughout history, these kinds of pandemics not only affected infected individuals but also entire societies in numerous ways: economic, social, psychological, and physical [3]. Despite warnings that had been around for tens of years, many countries were unprepared for the Covid-19 pandemic. While some managed to effectively control it, others failed [4]. Its other effects on health and social life will probably only be understood in the long term [4]. By end of July 2021, approximately 4.1 million people lost their lives due to Covid-19.

## 2. Isolation effects 

One of the most frequently used and historic methods for controlling pandemics is imposing quarantines. Depending on the type of the disease, there could be slight differences in the implementation of a quarantine; it is a dynamic process that requires regular updates according to changing conditions and selected pathology method. On the one hand, isolating people during these hard times provides many benefits, while it can cause some undesired personal and social outcomes. Due to Covid-19’s mode of transmission, terms such as quarantine, isolation, social distance, etc. have been a part of our daily lives. In the article titled “A Desperate Plea”, Richard Horton mentions how an important aspect of the pandemics has been underestimated [5]. This text points out that while focusing on macroscopic aspects of the pandemics, microscopic effects should not be disregarded as these tend to exacerbate hopelessness, fear, and stress among the society [5]. This is very important as the paramedical side effects of the pandemics should not be forgotten. Many studies have shown that isolation negatively affects the patient’s care and satisfaction. There are two overarching reasons behind these negative effects, one is patient-related and the other is healthcare worker-related [6, 7]. Abad and his colleagues, in an extensive review that they prepared with data spanning a period of 43 years, showed that isolated patients were more negatively affected in terms of psychological wellness, satisfaction, and feeling safe [6]. Furthermore, studies that evaluate pandemics, including Covid-19, and asses the psychological wellbeing of the health workers, report that health workers tend to minimize contact with their patients. It has been hypothesized that this stems from a lack of accurate information among health workers about the topic and the fear of contracting the disease from patients and transmitting it to their families [6, 8]. Therefore, disruptions to the routine healthcare provisions and a decrease in overall healthcare quality are expected. To minimize such negative outcomes, there are some suggestions and strategies [9]. New digital technologies can support treatment and care processes in both hospitals and the region [10]. The regular control of patients’ vital parameters via wearable technologies and smartphones could guarantee better management of health services [9]. Additionally, technological advancement can allow remote control of some medical devices, thereby minimizing contact between medical staff and infected patients [9, 11].

## 3. Overuse of antibiotics

Historically, one of the reasons why pandemics lead to devastating outcomes has been the inability to find a treatment during the acute phase of the disease. Currently, there is no effective treatment against Covid-19 pneumonia caused by SARS-CoV-2 TCSBHSGM. COVID-19 (SARSCOV2 Enfeksiyonu) Rehberi (Bilim Kurulu Çalışması) 2020 [Available from: https://covid19bilgi.saglik.gov.tr/depo/rehberler/COVID-19_Rehberi.pdf?type=file.. The absence of an effective treatment for the disease can lead to the inappropriate use of antibiotics [12, 13]. It is an irrefutable truth that antibiotics are useless in viral pneumonia. In the treatment guidelines prepared by our ministry of health, antibiotic treatment is only suggested when there is a suspicion of bacterial pneumonia^1^. The rising pressure on health systems due to the pandemic, difficulty in Covid 19 diagnosis, and doctors’ tendencies to not take risks decreased the adherence to the guidelines. Globally, the situation was no different. While early deescalation attempts are recommended, the high workload in emergency rooms and microbiology laboratories, the absence of an evidence-based treatment of Covid-19, the stress factor on doctors, and the antibiotic presence in the WHO guidelines for the disease caused a rise in antibiotic use rates World Health Organization. Statement on the second meeting of the International Health Regulations (2005) Emergency Committee regarding the outbreak of novel coronavirus (2019-nCoV). 2020 [Available from: https://www.who.int/news-room/detail/30-01-2020-statement-on-the-second-meeting-of-the-international-health-regulations-(2005)-emergency-committee-regarding-the-outbreak-of-novel-coronavirus-(2019-ncov) [12, 13]. The use of antibiotics in SARS-CoV-2 patients is significantly higher than bacterial coinfection and secondary infection incidences. According to metaanalyses, 7%–8% of the patients who are diagnosed with Covid-19 have developed bacterial or fungal infections [14]. In the patients in intensive care units (ICU), this rate is higher (8%–14%). Only 3.5% of the patients had co-infections while 14.3% had secondary infections [15, 16]. Although bacterial infection notification rates were low, antibiotic use in Covid-19 patients was quite high, and 71.9% of these patients took antibiotics; 74% of the prescribed antibiotics were fluroquinolones and third-generation cephalosporins [15]. For the appropriate use of antibiotics in Covid-19 patients, it is important to classify patients as being either ventilated-critical, hospitalized but not ventilated, or outpatient. Thus, proper criteria for each group will open a door to smarter antibiotic use [17]. In a recent study, there were low co-infection incidences in critical patients, but the infections tended to develop after hospitalizations [18]. On the other hand, although detailed data is missing, a first look at the data showed us outpatient antibiotic use may be diminished in some places either due to lockdown effects on other diseases’ transmissions or the limitations on healthcare services or a combination of both [19]. However, antibiotic use in many Covid-19 patients globally increased in comparison to the pre-pandemic period [12, 20, 21]. There is a need for more studies to assess the possible negative effects of using disseminated antibiotics. The expected result is that antibiotic resistance increases as an outcome of increased antibiotic use. Covid-19 pandemic’s effect on antimicrobial drug resistance is still not fully understood [19, 22–26]. Because the mode of SARS-CoV-2 transmission requires more attention on hand hygiene and use of personal protective equipment (PPE), these could have positive effects on antimicrobial drug resistance [27, 28]. On the other hand, an uncontrolled rise in the number of patients and difficulty in reaching PPE can motivate multidrug resistant bacteria to manifest [27–29]. As the pandemic grew, the staff members and resources used in monitoring antimicrobial drug resistance were directed towards diagnosis and the follow up of Covid-19 patients. Including anti-microbial drug follow ups, all of the non-Covid-19 studies were interrupted or dropped. As countries applied international travel restrictions, the info sharing and activities to build up international networks were disrupted [17]. To maintain antimicrobial drug resistance research, they should be financially supported and efforts to sponsor these activities should be prioritized. It is important to ensure current sample collecting and studies on diagnosis tools are being developed in order to support activities regarding antimicrobial drug resistance [17]. It is necessary to develop standardized protocols for Covid-19 related co-infections and secondary infections under the mentorship of WHO researchers. If implemented correctly, data flow between research centers would be guaranteed [30]. Resistance follow ups in both Covid-19 and non-Covid-19 patients should continue [31–34]. Epidemiological data from colonized patients, high risk patients, or infected patients should be collected. In high risk areas (such as ICUs and hematology departments) and especially when cohorting is used in Covid-19 patients, active surveillance for colonization in high risk patients should continue.

## 4. Problems in chronic disease follow-ups

### 4.1. Patients with cardiovascular diseases

As the pandemic grew dramatically, Covid-19 patient load led to capacity problems in emergency rooms and ICUs; it also affected the availability of PPE and led to problems in medical staff sufficiency. Because of this, medical institutions limited or postponed elective operations and lowered the patient care. On the other hand, management of the patient groups who required urgent or regular care was deemed a medical emergency that had to be orchestrated amongst the challenges of the Covid-19 pandemic. Cardiovascular diseases are an example of these kind of diseases. One third of worldwide deaths is caused by cardiovascular diseases. The management of the acute myocardial infarction is important in this Covid era. Although there is a link between cardiovascular diseases and Covid-19, most of the patients who need cardiovascular care may not be infected with this virus [35]. Clearly, it is a hard task to give recommendations for acute myocardial infarction patients during a pandemic. Considering the risk of Covid-19’s asymptomatic spread, some cardiovascular signs of Covid-19 might be confused with various coronary emergencies, and this makes management of these patients even harder [35]. In some regions Covid-19 spread among the society is high and sensitivity of the test is problematic [31, 35–37]. In addition, some positive tested patients can be asymptomatic although the findings are present in computerized tomography. It must be known that asymptomatic spread rate is high [36, 37]. For this reason, without taking the Covid situation into account, it is important to properly assess patients who need invasive intervention, making sure medical staff who could be exposed to the virus are safe and that contamination risk during cardiac catheterization is minimized. In the guidelines prepared by Mahmoud and his colleagues, there is a systematic approach for patients who experience acute myocardial infections during the Covid-19 era [35]. According to these guidelines, the first goal should be taking measures to minimize Covid-19 exposure among the society, thus guaranteeing that patients who show up with acute ischemic cardiac disease symptoms continue to access medical services without interruption [35]. In indicated patients, percutaneous coronary intervention and fibrinolytic use should not be avoided. Patients who undergo intervention must be wearing masks and PPE in order not to endanger healthcare workers [35]. In some conducted studies, it was found that there are prolongations between acute cardiac symptom onset times and hospital arrival times [38]. This can lead us to think that, during the pandemic, there could be some difficulties to reach medical care. Since everyone primarily focuses on respiratory symptoms during pandemic, some misdiagnoses could occur, and the quality of medical care could suffer [15]. It is important to develop effective strategies for cardiac emergencies and simultaneously make sure both of these important emergency situations, Covid-19 and acute cardiac problems, are being properly managed. 

### 4.2. Patients with chronic kidney disease

Chronic kidney disease is a public health issue that affects 850 million people worldwide [39]. Chronic kidney disease patients are generally elderly people with many comorbidities [40]. Some of these may require immunosuppressive treatment. Patients who need regular dialysis are also at high risk of contamination during the pandemic [41]. Taking all of these factors into consideration, we can say that chronic kidney disease patients are more prone to get infected with Covid-19 in comparison to the general population [40, 41]. Furthermore, contracting Covid-19 can result in sudden kidney function test disorders and even death [40]. In order to mitigate the pandemic’s destructive effects, experts in this field gathered and published suggestions [42, 43]. These suggestions can be grouped into three categories: ones for health care providers, ones for chronic kidney disease patients, and ones for patients who take dialysis and dialysis centers [42]. Healthcare providers should mainly be giving attention to ensuring that the staff who will be involved in these patients’ treatments and care are wearing masks and other PPE. In addition, healthcare providers should avoid crowded meetings and activities. On the other hand, the staff working in healthcare institutions should be able to follow up on their or their patients’ symptoms and notify the responsible people when necessary [41, 44, 45]. Chronic kidney disease patients should avoid close interactions with others, and treatments prepared for these individuals should be under close supervision of their doctors. Also, as long as it is possible, visits to hospitals should be minimized, and online platforms should be promoted [41, 44, 45]. In dialysis units, traditional measures should be taken; Covid-19 suspected patients should have an isolated place separate from other patients. Also, dialysis should be as late as possible during the day for those who are infected with Covid 19. It is important that all the patients wear masks during the dialysis as well [41, 44, 45]. Everyone must follow the rules and medical protocols closely and completely in order to ensure that these sensitive groups of patients are minimally affected during this Covid-19 era.

### 4.3. Patients with malignancy 

Another group of patients who require special treatment, follow ups, and healthcare are patients with malignancies. This group of patients are susceptible to Covid-19’s effects, and it is essential for these patients to acquire health services under carefully planned measures. Nevertheless, the pandemic pushed many oncologists to change their work routines. This negatively affected patients follow ups and care as well as numerous cancer studies [46]. The dependable health protecting measures taken among the public in order to limit the virus’s spread has caused a slowdown in many institutions, including cancer research laboratories [46, 47]. Scientific experimental research requires sustained work, and it necessary in order to provide the best care to cancer patients. Many centers tried to change their programs without interrupting the studies and adapted to the new conditions [46]. On the other hand, some cancer research laboratories directed their efforts (in antineoplastic agents) towards researching therapeutic options for Covid-19 infections [46, 48]. The following are some points oncologists underline in their cancer research: 1) not leaving behind the patients whose survival and prognosis are dependent on a research treatment, 2) minimizing unnecessary exposure, and 3) reducing the pandemic’s exposure curve and increasing patients’ and research teams’ motivations [49]. As pandemic durations became prolonged, the disease progression risk in these patients left the Covid-19 infection risk from hospitals behind [50]. Due to this reason, experts in this field worked to prepare the best protocols in order to provide a balance between managing these sensitive patient groups and their protection from the virus. As the pandemic continued, different oncology organizations kept publishing suggestions and various guidelines regarding the management of these sensitive patient groups [51]. However, in spite of these efforts, it is hard to suggest optimal healthcare suggestions for these patients. Many guidelines emphasize that the approach to cancer patients should be dynamic and differ from patient to patient, hospital resources, and doctors’ personal experiences [52, 53]. Oncologists should keep in mind that, if the Covid-19 pandemic escalates, the risk of not having high-level medical care for cancer patients will be greater than the SARS-CoV-2 infection risk for those patients [46, 54]. The primary strategy was stopping or postponing palliative care for poor prognosis expected patients and giving priority to emergencies for aggressive diseases [55]. Also, protecting the medical staff was important for patient safety. Kimmig and his colleagues emphasized that choosing laparoscopic procedures in oncological surgery is important to protect medical staff by minimizing their exposure to aerosols [56]. Carbon dioxide gas management and appropriate filter use was brought into attention [57]. In some centers screening facilities and oncology services were re-designed to provide safer accommodations for patients [58]. Most of the doctors preferred non-surgical invasive procedures in cases that did not necessarily require surgery, as fecal and oral transmission of the virus had not been completely ruled out. Also, many surgeons encouraged the avoidance of endoscopic procedures [59]. For medical treatment, it was generally recommended to decrease adjuvant chemotherapy frequency and density [60]. Some clinics formulated algorithms according to the specific situation in their respective countries. In Italy, where the destructive effects of the pandemic have clearly been seen, a hospital suggested an algorithm for uro-oncological operations where they classified the patients. Cases like muscle invasive bladder cancers or locally-advanced prostate cancers were not to be delayed while some cases were half undelayable and some others are delayable. Another category was the group of diseases in which other nonsurgical treatments could be an alternative to surgery [53].

## 5. Mental health

Emergency situations that concern public health, such as pandemics and wars, also closely affect social security, health, and prosperity. These can have influences on mental health as well. Studies dealing with emergency cases’ effect on mental health have proposed that emotional effects can be present in every layer of society [61]. The case of Covid-19 is no different. Some groups are more vulnerable to the pandemic’s psychosocial effects. One of the most important groups here are healthcare workers. Healthcare workers generally work in the front lines in such emergency cases. Mental wellbeing among healthcare workers would also have globally positive effects on social recovery along with controlling the effects of the pandemic [62]. An increasing number of cases, absence of efficient drugs and treatment protocols, inadequate amounts of PPE, misinformation in media, increasing numbers of Covid-19 related deaths among healthcare workers, and not having sufficient psychosocial support are some of the reasons behind the increasing psychological load among healthcare workers [63, 64]. Medical staff did not know much about Covid-19 since it began suddenly and spread quickly. They also were not mentally prepared for this big disaster. This caused psychological trauma at different levels [65]. Depression, anxiety disorders, and insomnia are some of the psychopathological problems among healthcare workers [61]. In the conducted studies, it has been shown that, during pandemics, negative psychological reactions are becoming more prevalent among healthcare workers [66, 67]. Studies showed that healthcare workers were in fear of transmitting the disease to people they are close to, and they are felt desperate about the pandemic’s progress and often thought of leaving their jobs [66, 67]. Besides these factors, they were also experiencing high levels of stress, signs of anxiety, and depression [68]. Post-traumatic stress disorder is a condition that more likely occurs after natural disasters, technologic accidents and attacks, and mass-destruction attacks. It is characterized by mental imbalance following psychological trauma [61]. Long lasting disasters are more often linked to post-traumatic stress disorders in comparison to short lasting disasters. So, it is possible to say that pandemics cause qualitatively and quantitatively more serious effects on healthcare workers when compared to short lasting issues [69, 70]. Findings that post-traumatic stress disorder rates are higher after the Covid-19 and SARS pandemics actually showing healthcare workers are sensing those as disaster [71–74]. The study regarding post-traumatic stress disorders and their affiliated insomnia problems, conducted by Bulut and his colleagues, had some important discoveries. This study was also meaningful since it was conducted in the Van province where the very first suspected cases were seen and the first pandemic hospital was designed. According to study, posttraumatic stress disorder symptoms were seen in high rates in all healthcare workers [63]. These symptoms were more frequently found in women. According to the classification based on jobs, nurses, especially those who are married, were affected the most. In other related studies, similar results were obtained. It was noted that nurses are in more contact with patients during the day, they are mostly women, they do not know about the disease as much as doctors, and they were more likely to internalize the problems they faced and present more psychological symptoms [64, 65, 71, 74–77]. It was also demonstrated that married healthcare workers worried not only about themselves but also their families and children [63, 72]. These kinds of studies are important to assess which group of healthcare workers are being affected more. Among the healthcare workers, focus should be on the groups who carry more psychosocial risks, and these groups should be prioritized in psychoeducation, psychological support, and screening for mental problems. In this process, a mobile application called “RUHSAD” was created by the Ministry of Health of the Republic of Turkey in order to protect and support the mental health of healthcare professionals and their children. With this application, healthcare professionals and their children were allowed to make video calls with specialist psychiatrists. In these video calls, priority was given to those who were in quarantine due to Covid-19 whose test results were positive, whose relatives were permitted in the ICUs, and who were assigned to the Covid-19 units https://ohsad.org/saglik-bakanligi-saglik-hizmetleri-genel-mudurlugu-tarafindan-ruhsad-uygulamasi-hakkinda-duyuru-yayinlandi/. Published 2020, [accessed June 12, 2021]. The measures that are being taken during a pandemic should be multilateral. Besides this, protecting these healthcare workers may be the most important measure that could be taken towards Covid-19 and its effects on public health. Fighting this pandemic with mentally powerful healthcare workers is an essential component to winning this fight. Interventions to prevent mental problems and develop strong mental health among the healthcare workers fighting Covid-19 in front lines should be applied in early period, and risk groups should be strongly supported.

## 6. Taking social aspects of the pandemics into account and making efforts to minimize harm to people during global health crises

A comprehensive approach by those in authority would help overcome these difficult times. Coordination and collaboration among the authorities will lead to strong and consistent responses to such a crisis. This, at the end, will produce benefits to people struggling with the proposed process. In this regard, the multilateral approach to health topics that started in Turkey in 2011 has been an important step. It can be a solid foundation as Turkey properly takes care of its people during pandemic and shows efforts to overcome the pandemic. During the Covid-19 pandemic, it is important to be prepared in health and health system aspects as well as for the social side of the problem. For this purpose, a project, “COVID-19 ve Toplum: Salgının Sosyal, Beşeri ve Ekonomik etkileri, Sorunlar ve Çözümler” (COVID-19 and Society: Pandemic’s Social, Sociological and Economic effects, Problems and Solutions) has presented some results. According to the study, Covid-19 fear, obsessive compulsive symptoms, health worries, negative feelings, and sleep quality have played important roles in the pandemic in relation to citizens’ psychological statuses. At this point, cognitive-behavioral approaches may be beneficial for society while measures to assist sociological mental health are being taken Yalçın İ. Siberkondri, COVID-19 Korkusu, Sağlık Kaygısı, Obsesyonlar, Uyku Kalitesi ve Duygulanım: Bir Karışımlı Yapısal Eşitlik Modellemesi Yaklaşımı. https://www.tubitak.gov.tr/sites/default/files/Covid19veToplum/ozet-ozgecmis/23SUBAT/FEZAGURSEYSALONU/10.00-11.30/IlhanYALCIN_Ozet.pdf. Published 2021, [accessed June 12, 2021]. It is also thought that online or mobile phone-based psychological education and interventions might be an alternative to support people. In our country, especially due to mental problems, people do not regularly seek help in psychiatry departments in hospitals. Mobile phones and online tools could be suitable alternatives to help to cover the need. In this context, mobile-based self-help intervention services and providing free access to all would be a good method to combat the problem. It is suggested that, while new ideas are arising, these methods should be amplified by using technology and modern solutions İnözü Mermerkaya M HAB. COVID-19 Pandemisine İlişkin Ruh Sağlığı Çalışmaları: Pandemi ile İlişkili Psikolojik Zorlanmaların Değerlendirilmesi, Mobil Telefon Temelli Uygulamaların ve Web Temelli Psiko-Eğitim Paketlerinin Geliştirilmesi. Covid-19 ve Toplum, Salgının Sosyal, Ekonomik ve Beşeri Etkileri, Bulgular, Sonuçlar ve Öneriler. https://tubitak.gov.tr/sites/default/files/20689/covid_19_ve_toplum_salginin_sosyal_beseri_ve_ekonomik_etkileri_sorunlar_ve_cozumler.pdf. Published 2021, [accessed June 12, 2021]. In Turkey, a study with 3004 participants with ages ranging from 18 to 75 in 76 different cities has been carried out to stratify risk groups by evaluating participants’ anxiety levels, depression, somatization, hostility signs, and demographic parameters. This study reveals that women, single individuals, people below 30 years old, people with lower income and education levels, people who are diagnosed with at least one psychiatric disorder, and people who have close relatives affected with Covid-19 have developed more psychological symptoms and signs. It is assessed that mass communication tools and online platforms should be used more effectively and should be primarily considered while intervention plans are being developed for these target groups Haciömeroğlu A. B İMM. COVID-19 Salgını ile İlişkili Psikolojik Belirtilerin Görülme Sıklığı: Belirtilerin Şiddetlenmesi ile İlişkili Risk Faktörleri ve Koruyucu Faktörler . 120K408. Covid-19 ve Toplum, Salgının Sosyal, Ekonomik ve Beşeri Etkileri, Bulgular, Sonuçlar ve Öneriler. . https://tubitak.gov.tr/sites/default/files/20689/covid_19_ve_toplum_salginin_sosyal_beseri_ve_ekonomik_etkileri_sorunlar_ve_cozumler.pdf Published 2021, [accessed June 12, 2021]. In another study, the intrepidity experiences of people who contracted Covid-19 and recovered were mainly classified into four groups: 1-Preventive factors, 2-Risk factors, 3-Positive outcomes, 4-Feelings. The main preventive factor was social support, and this clearly suggests that more efforts need to be shown in order to improve relations with friends, families, and relatives. This may be through remote communication using mobile phones, laptops, and any other current technological tools. Inner preventive factors, such as hope and optimism, can help and accelerate medical treatment success and aid in producing positive outcomes. Risk factors include social isolation, interruption in education, and economic difficulties. Positive outcomes include improvement with family relationships, discovering new possibilities for selfadvancement, and having a new viewpoint towards life Karaman M A Ar, Tomar İ H, Eşici H, Özbay Y. COVID-19 Pozitif Geçmişi Olan Bireylerde Ruh Sağlığını Koruyucu Faktörlerin Belirlenmesi. Covid-19 ve Toplum, Salgının Sosyal, Ekonomik ve Beşeri Etkileri, Bulgular, Sonuçlar ve Öneriler. https://www.tubitak.gov.tr/sites/default/files/Covid19veToplum/ozet-ozgecmis/23SUBAT/ASALONU/14.00-15.00/MehmetAkifKARAMAN_Ozet.pdf , [accessed June 12, 2021]. In another study that dealt with Covid-19’s effects on children and adolescents, as well as their mothers in terms of their cognitive- psychosocial improvement and academic life, it was documented how difficulties that children face through distant education also negatively affect their mothers. Mothers were found to have more depression and higher stress levels in comparison to pre-Covid times Berument Sk Da, Acar B Ş, Tahiroğlu D. COVID-19’un Çocuk ve Ergenlerin Bilişsel ve Psikososyal Gelişimi ile Akademik Hayatına Etkileri. Covid-19 ve Toplum, Salgının Sosyal, Ekonomik ve Beşeri Etkileri, Bulgular, Sonuçlar ve Öneriler. https://tubitak.gov.tr/sites/default/files/20689/covid_19_ve_toplum_salginin_sosyal_beseri_ve_ekonomik_etkileri_sorunlar_ve_cozumler.pdf [accessed June 12, 2021]. 

## 7. Overcoming the pandemic

Vaccine development has never progressed this quickly and successfully. Conventional vaccine development time used to be approximately 10–15 years; during the Covid-19 pandemic, this duration has been decreased to a remarkable 12–18 months. In order to make this achievement, the time required for each step of the vaccine development process has been shortened. At the same time, parallel work has been implemented, and approval times for developed vaccines has been shortened. Some countries contributed financially to this quick vaccine process. We also have to note that previous work, which focused on MERS-CoV and some cancer vaccines, has helped in the accelerated development of the Covid-19 vaccine. The accumulated information was channeled in the context of this new pandemic. Taking all of these factors into account, these vaccine studies provided the expected result in record time. It is important to note, again, that applying emergency approval procedures for vaccines also aided these vaccines to be immediately put in use. Accelerating factors such as these should always be considered with any given potential global health problem; however, it is still essential to properly clarify them and their boundaries. Easy access to data and new information, mutual budgets for further researches, optimization of early approval processes, determining the sustainable rules for copyrights issues, and optimization of the global approach towards personal and political interests, as well as everyone’s right to access health services fairly, are crucial. 

## 8. Increasing access to vaccines and patent rights issues

Patents serve as the recognized copyright of any product or service that has been implemented, providing legal protection for inventors. It has a long history going back to the 1400s; since then, this process has been revised and developed many times. Practically, a patent offers three important benefits:

1-It serves as a motivator for new research and future inventions;

2-It provides merriment, prestige, and economic advantages to inventors—so, new research and projects are always underway;

3-By protecting new technologies, it allows patent owners to invest in different countries, thereby spreading the technology or idea [78].

In the context of vaccines, a patent may include vaccine formulations, any other substances added to formula, automatic injectors needed for its application, nasal application tools, capsule formulations, and many other related things. During this process, conditions for quality and control issues and transportation requirements may occur. In addition, trademark rights and secrets regarding the process would be protected; know-how topics are also included here. One of the most important functions of patent rights in vaccine production is motivating sponsorship in research and development investments. If no financial support is provided, development in this field cannot be expected. A quality control system is another critical step here, especially in the sense of effectiveness and quality. Public acceptance is a key point in vaccination program success. This is valid for any vaccine Stevens H, Debackere K, Goldman M, Mahoney RT, Stevens P, Huys I. Vaccines: accelerating innovation and access. *Global Challenges Report, World Intellectual Property Organization (WIPO). *2017. https://www.wipo.int/edocs/pubdocs/en/wipo_pub_gc_16.pdf [accessed June 12, 2021]. To end the Covid-19 pandemic, it is essential to deliver effective vaccines to all countries and all social groups within them. There are some barriers for broad access to vaccines, such as vaccine development, production, distribution, implementation, and acceptance by people as well as follow ups during the postvaccination period. All of these steps must build off one another smoothly to determine that the vaccination program is successful. Unless all these steps are successfully carried out, it is useless to sacrifice patent rights and expecting all the obstacles to spreading the vaccination program to disappear. Together with sacrificing patent rights, convenient infrastructure for production, and know-how transference, proper quality control processes should be completed. Vaccine accessibility can be improved though a tiered pricing system that is already in use for many vaccines. For the potential new pandemics that can spread widely and require new drugs/vaccines, vaccine development and legal studies to build a strong base for those efforts should continue. New regulations and ideas will better help vaccine accessibility worldwide and motivate people who are dealing with this important subject. Although the last pandemic deeply affected almost every country (i.e. the Ebola virus epidemic in 2014) countries with poor health infrastructures are more heavily impacted. To combat infectious diseases and their effects such as the one we are fighting now, it is important to fortify health systems and recruit human sources in the current medical system. Human resources in the medical field should be planned to effectively preserve both quality and quantity. While clarifying missions for each branch, it is also necessary to retain vocational training TC Sağlık Bakanlığı Halk Sağlığı Genel Müdürlüğü. Çok Paydaşlı Sağlık Sorumluluğunu Geliştirme Programı, Tedavi ve Rehabilite Edici Sağlık Hizmetlerinde Çok Paydaşlı Yaklaşım. Sağlik Hizmet Sunumunda İnsan Kaynaklari Yönetimi. T. C. Sağlık Bakanlığı Türkiye Halk Sağlığı Kurumu, Bakanlık Yayın No: 1003. https://hsgm.saglik.gov.tr/tr/cevresagligi-suguvenligi/su-güvenliği-ve-kaplıcalar-birimi/çok-paydaşlı-sağlık-sorumluluğunu-geliştirme-programı.html [accessed June 12, 2021]. In case of extraordinary situations, such as pandemics, we may need to re-organize the healthcare system’s human resources without totally neglecting their primary fields of responsibility.

## 9. Being ready

There are pandemic plans prepared and regularly updated by WHO as well as by various governments. If these plans are regularly being regulated and updated, it would allow the governments to take better action in a timely manner and increase their capacity to fight pandemics. This has been tested during Covid-19 too. Turkey had updated its influenza pandemic plan in 2019; taking some similarities between Covid-19 and influenza into account, this allowed Turkey to react properly and quickly to the Covid-19 pandemic World Health Organization. Essential steps for developing or updating a national pandemic influenza preparedness* plan.* World Health Organization;2018. https://apps.who.int/iris/bitstream/handle/10665/272253/WHO-WHE-IHM-GIP-2018.1-eng.pdf?ua=1 [accessed June 12, 2021]. The Covid-19 pandemic has caused an unforeseen and complex situation in global health institutions and countries. An effective response to the pandemic required alternative approaches for the institutions who provide health services. The Covid-19 pandemic is an example of how a small and surprisingly isolated incident somewhere in the globe can actually have a huge influence on how medical practices are performed. This surprising side could be summarized in three titles: complexity of its root, the rate or spread, and the unpredictability of the pandemic`s effect. Health service providers had common shared problems as well as unique ones. Some of these were the need for PPE, patient bed capacity, intensive care unit bed capacity, ventilator needs, and the need for enough medical professionals. To solve these problems, institutions and countries reorganized themselves quickly. This reorganization process included reforming treatment and patient care models, keeping the medical professionals’ motivation and abilities high, and lowering their levels of anxiety. Another issue has been that of financial sustainability of hospitals and maintaining services for non-Covid-19 patients. Additionally, in the future, if a disaster like a terrorist attack or another pandemic occur, health institutions will have to arrange themselves according to the new state. During the Covid-19 pandemic, international organizations as well as national and regional institutions have gained the flexibility to immediately adapt to any new and unforeseen situations. This could be seen as an opportunity in this context World Health Organization. World Health Organization coronavirus disease (COVID-19) dashboard. 2020, https://www.who.int/emergencies/diseases/novel-coronavirus-2019 [accessed June 12, 2021] [79,80]. When the very first cases were announced in China, while not many cases were seen in Turkey, a national advisory committee was formed. This committee had valuable insight and quickly suggested guidelines for patient care. These guidelines have been used in hospitals, to inform the public and for the medical personnel [81]. Here are some of those guidelines that have been used to educate medical professionals, as seen in Table.

**Table:** Main guides for health care professionals during COVID-19.

Main guides	Content
Working guide and infection control measures	In health institutes, how health care professional work and what kinds of measures should be taken are all explained in this guide.
COVID-19 (SARS-CoV-2 INFECTION): General information, epidemiology and diagnosis	In this guide, the definition, epidemiology and case process (case detection, case tracking and case management) are explained.
Adult patient treatment	This guide explains the process of adult patient treatment.
Child patient management and treatment	It includes child patient management process and treatment process during COVID-19.
Antitoxin, antiinflammatory treatments,coagulopathy management	It includes main recommendations for the treatment of hyperinflammatory and the management of coagulopathy during COVID-19.
Contact monitoring, outbreak management, monitoring patient at home and filiation	It explains the process of contact monitoring excluding health care professionals. This guide also includes pandemic management, filiation process, and monitoring patient at home.
Infection control and isolation	It includes the process of infection control and isolation.
Morgue and burial services	It includes process of morgue and burial services through main measures during COVID-19.

(*) Adapted from reference 81.

## 10. Resilience to other potential pandemics

In most of the international organizations and developed countries, regular practices for catastrophic situations are being made. It is not a single decision, rather these must be made on a regular basis in order to keep the necessary systems alert and prepared for such crises. These practices are implemented through a viable scenario and not based on unreal fictions. Scenarios are based on real problems that are not present at the moment TCSBTHS Kurumu. Çok Paydaşlı Sağlık Sorumluluğunu Geliştirme Programı, Tedavi ve Rehabilite Edici Sağlık Hizmetlerinde Çok Paydaşlı Yaklaşım. Acil durum ve Afetlerde sağlık hizmetlerinin organizasyonu . T. C. Sağlık Bakanlığı Türkiye Halk Sağlığı Kurumu. Published 2016.. https://hsgm.saglik.gov.tr/tr/cevresagligi-suguvenligi/su-güvenliği-ve-kaplıcalar-birimi/çok-paydaşlı-sağlık-sorumluluğunu-geliştirme-programı.html [accessed June 12, 2021]. Some different disasters, such as pandemics, may force us to make preparations with limited timeframes. However, most of the time crisis plans are still useful, at least in early phases of pandemics. It gives us a chance for re-organizing ourselves and our long term efforts. Considering this, national crisis plans should be worked on in advance. Since the Almaty declaration in 1978, health and multisectoral approach have been going forward together. Terms such as Sustainable Development Goals (SDG) and One-Health concept and Health in All Policies (HiAP) are building a base for a multisectoral approach. Social determinants have come into prominence for health alongside these processes World Health Organization. Health in All Policies as part of the primary health care agenda on multisectoral action. World Health Organization; 2018. https://www.who.int/publications/i/item/WHO-HIS-SDS-2018.59 [accessed June 12, 2021]. For health systems to manage crises and be successful, they should be properly orchestrated. To maintain the durability of the health system, we have to place health and related issues in the center of global policies, as seen in Figure. 

**Figure:**
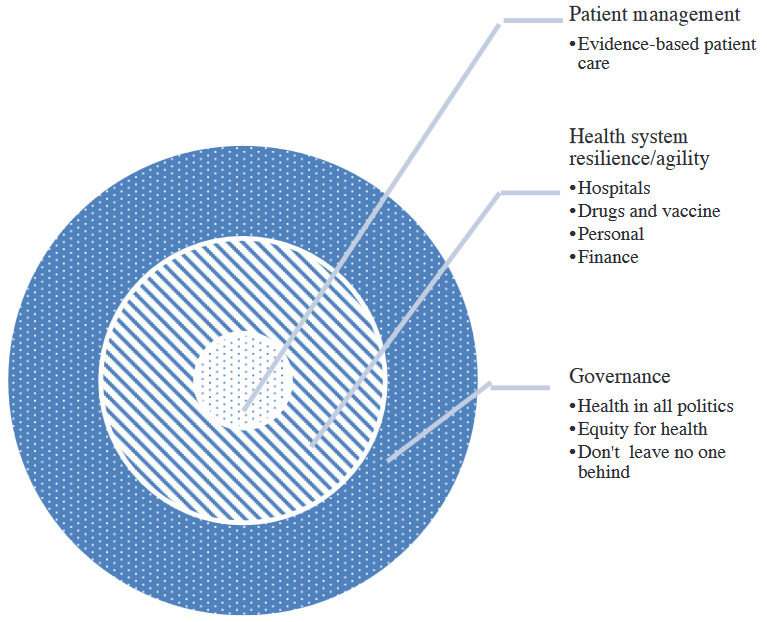
Durable and ready for crisis in health systems.

As countries’ practices of multisectoral approaches develop, we can expect health crises such as pandemics that have disastrous effects to be easier to overcome. As HiAP concept and leave-no-one-behind approach are embraced more readily, we could have a better chance in our fight with the pandemic. The Covid-19 pandemic showed us that it is useless to group health services into preventive and treatment services or infectious diseases and noninfectious diseases [82]. Organizations giving priority to maintaining global health security and universal health coverage, together in balance without needing external funds, are more likely to be successful in the Covid-19 era in comparison to fragmented health institutions [83]. Placing the health topics in the center of all policies helps in building a new health system concept that will help us to have better results in improvement efforts. This will also help minimize future pandemics’ destructive results.

## 11. Conclusion

The Covid-19 pandemic time has been a test for national and global health institutions’ capacities to fight such crises. Having observed failures alongside partly- and fully-successful examples, we now have ideas for possible upcoming crises. At the same time, for complete success, it has been clear that no one should be left behind; this approach is mandatory. Although there are many plans, guidelines, and maneuvers prepared by national and global organizations, in practice, things might be different in the moment of crisis. For these to be practiced easily, information must be flexible and internalized by all acting parties and their supporters, because no crisis will be the same as a previous one. Resilience, alongside flexibility and quickness, is critical in long lasting and large scale disasters like Covid-19. In order to achieve this level of resilience, we need global policies that critically consider physical and mental health practices as well as health system policies.
